# Antimicrobial Power of Organic Acids and Nature-Identical Compounds against Two *Vibrio* spp.: An In Vitro Study

**DOI:** 10.3390/microorganisms9050966

**Published:** 2021-04-29

**Authors:** Barbara Rossi, Maria Angéles Esteban, José María García-Beltran, Giulia Giovagnoni, Alberto Cuesta, Andrea Piva, Ester Grilli

**Affiliations:** 1 Vetagro SpA, 42124 Reggio Emilia, Italy; barbara.rossi@vetagro.com (B.R.); andrea.piva@unibo.it (A.P.); 2 Fish Innate Immune System Group, Department of Cell Biology and Histology, Faculty of Biology, University of Murcia, 30100 Murcia, Spain; aesteban@um.es (M.A.E.); josemaria.garcia4@um.es (J.M.G.-B.); alcuesta@um.es (A.C.); 3 DIMEVET, Department of Veterinary Medical Sciences, University of Bologna, 40064 Ozzano dell’Emilia, Italy; giulia.giovagnoni4@unibo.it; 4 Vetagro, Inc., Chicago, IL 60604, USA

**Keywords:** *Vibrio harveyi*, *Vibrio anguillarum*, organic acids, botanicals, antimicrobials, antimicrobial resistance, aquaculture

## Abstract

Vibrosis is one of the major threats in aquaculture farming, and due to the increasing antimicrobial resistance of different *Vibrio* species, there is an urgent need to replace conventional treatments with more sustainable solutions. Antimicrobial molecules such as organic acids (OA) and nature-identical compounds (NIC) are currently finding a central role in the infection management of terrestrial livestock, but little is known about their usage in aquaculture. The aim of this study was to perform a preliminary screening of the in vitro antimicrobial activity of a wide panel of OA and NIC against 2 *Vibrio* species characteristic of the Mediterranean area, *Vibrio harveyi* and *Vibrio anguillarum*, through minimal inhibitory/bactericidal concentration tests. The active principles that showed the best effective pathogen control were medium-chain fatty acids, sorbic and benzoic acid among OA and phenolic monoterpenoids (thymol, carvacrol and eugenol) and aromatic monoterpene aldehydes (vanillin and cinnamaldehyde) among NIC. These results showed how the usage of OA and NIC can open promising perspectives in terms of *Vibrio* spp. load control in aquaculture. Natural or nature-identical feed additives can make aquaculture production not only more efficient by reducing the need for medicated treatments, but also more sustainable.

## 1. Introduction

Aquaculture is an important fast-growing production sector for protein sources that are easily digestible and have a high biological value [1]. One of the major threats in aquaculture farming is the sudden outbreak of diseases, especially those caused by *Vibrio* spp. [2]. *Vibrio* spp. occur naturally in the marine environment and represent the major bacterial pathogens affecting fish and shrimp farming, leading to severe economic losses worldwide [2,3]. High disease rates in intensive farming rely on antibiotics and other supplements, especially in countries where regulatory limits may not be clearly defined or monitored closely [4]. In fact, the regulatory framework regarding the use of antibiotics in aquaculture is limited and differs greatly between countries [5]. The prophylactic use of antibiotics in aquaculture has been commonplace in the past and nowadays, due to the difficulty in treating individual affected finfish and shellfish, the metaphylactic use of antimicrobials to treat entire populations is still habitual [4]. A consequence of this indiscriminate use of antibiotics is the promotion of antimicrobial resistance (AMR) and several investigations have been conducted in different countries regarding antibiotic resistance in *Vibrio* spp. isolated from aquatic species [3,6,7,8,9,10,11,12,13,14,15].

**Figure 1:**
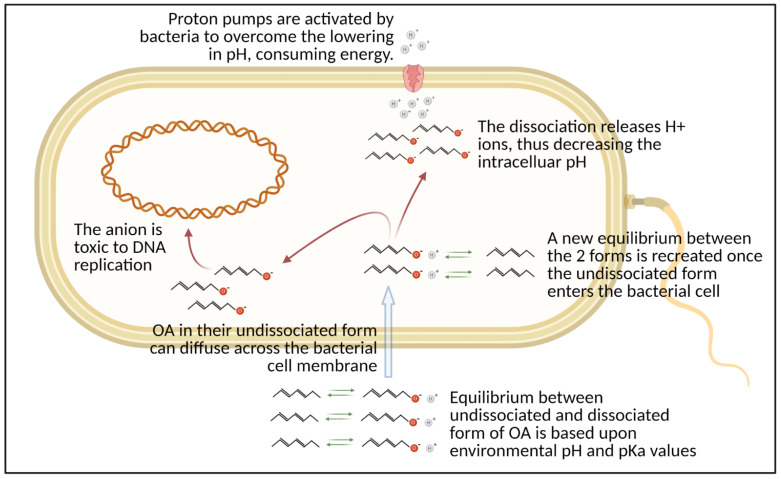
Antimicrobial mode of action of organic acids (OA). The anion model is generally accepted as mode of action for OA. Created with Biorender.com (accessed on 19 April 2021).

**Figure 2:**
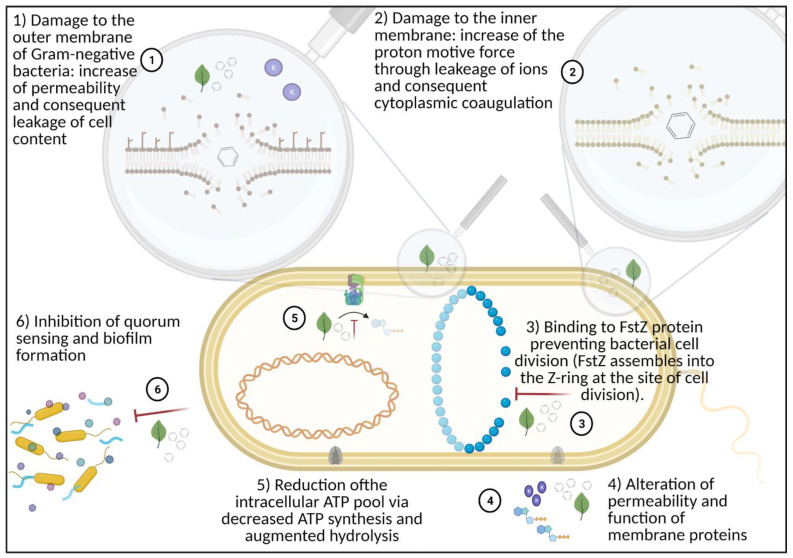
Antimicrobial mode of action of nature-identical compounds (NIC). Botanicals can have different targets to exert their antimicrobial mode of action (**1**–**6**). Created with Biorender.com (accessed on 19 April 2021).

In terrestrial livestock, different feed additives are used as an alternative to antibiotics with the aim to control the pathogen load [16,17]. The selection of these active compounds is based on their antimicrobial potential, as in the case of organic acids (OA) and botanicals [18,19]. Both OA and botanicals, in fact, have a well-recognized antimicrobial power, and the antimicrobial mechanism of action is different in relation to different classes of molecules [19]. OA action against microorganisms depends on the carbon chain length and degree of unsaturation, but overall, the pKa of the acid influences its antimicrobial mechanism of action (Figure 1). Botanicals include both plant extracts (composed of several bioactive compounds) and nature-identical compounds (NIC), the pure chemically- synthesized compounds identical to their counterparts present in plant extracts [18]. The antimicrobial mode of action of botanicals can have a single target or address multiple ones [20] (Figure 2).

The aim of this study was to perform a screening of the in vitro antimicrobial activity of a wide panel of NIC and OA against 2 *Vibrio* species characteristic of the Mediterranean area, *Vibrio harveyi* (*V. harveyi*) and *Vibrio anguillarum* (*V. anguillarum*). Ten OA and eleven NIC were selected among different chemical classes and according to their efficacy, previously detected against other terrestrial livestock pathogens [21]. More precisely, OA were selected among different classes such as short-chain fatty acids (SCFA—formic, propionic and butyric acid), medium-chain fatty acids (MCFA—hexanoic, octanoic, decanoic and dodecanoic acid), tricarboxylic acids (TCA—citric acid) and other OA widely used in the food and feed preservation (sorbic and benzoic acid). NIC were selected mainly among phenolic monoterpenoids (thymol, carvacrol, eugenol), other alcohol monoterpenoids (geraniol, menthol, linalool), cyclic monoterpenes (alpha-pinene, limonene, eucalyptol) and aromatic monoterpene aldehydes (cinnamaldehyde and vanillin).

## 2. Materials and Methods

### 2.1. Bacterial Strains and Growth Conditions

*V. harveyi* was isolated from diseased farmed gilthead seabream, while *V. anguillarum* was purchased from ATCC (ATCC 19264). Bacteria were grown under aerobic conditions at 25 °C in Tryptic Soy Agar (TSA, Difco Laboratories, Franklin Lakes, NJ, USA) and then inoculated in Tryptic Soy Broth (TSB, Difco Laboratories, Franklin Lakes, NJ, USA). Both were supplemented with NaCl to a final concentration of 1.5% (*w*/*v*).

### 2.2. Organic Acids and Nature-Identical Compounds—Chemicals and Test Solutions

The OA used were formic acid (For), propionic acid (Prop), butyric acid (But), hexanoic acid (Hexa), octanoic acid (Octa), decanoic acid (Deca), dodecanoic acid (Dod), citric acid (Cit), sorbic acid (Sor) and benzoic acid (Ben). The NIC tested were thymol (Thy), carvacrol (Car), vanillin (Van), eugenol (Eug), cinnamaldehyde (Cin), geraniol (Ger), alpha-pinene (aPin), eucalyptol (Euc), menthol (Men), linalool (Lin), limonene (Limo) and vanillin (Van). All the OA and NIC were purchased in pure form from Merck KGaA (Darmstadt, Germany). Stock solutions of OA (except Octa, Deca and Dod) were prepared in TSB supplemented with 1.5% NaCl (TSB + NaCl) and buffered to pH 6.5, whereas stock solutions of NIC, along with Octa, Deca and Dod, were prepared in 100% ethanol in order to increase their solubility before proper dilution in TSB + NaCl. Dilutions in fresh sterile TSB + NaCl were performed to reach the final concentrations tested and were buffered to pH 6.5 and filter-sterilized (Pore size 0.22 μm. Millipore Corporation, Billerica, MA, USA).

### 2.3. Minimal Inhibitory Concentration (MIC) Assay

The minimal inhibitory concentrations (MIC) of OA and NIC were determined using a broth microdilution method in 96-well microtiter plates [22].

The bacterial strains (10^5^ CFU/mL) were incubated in TSB + NaCl with the tested substances under aerobic conditions at 25 °C for 2, 4, 6, 8 and 24 h with continuous shaking (100 rpm). In case the stock solution of the substance tested was prepared in ethanol, as described in paragraph 2.2, control strains were grown in a medium containing 5% (*v*/*v*) ethanol, to exclude the possibility of any inhibitory effects mediated by ethanol. In fact, 5% (*v*/*v*) was the highest amount of ethanol eventually brought by stock solutions prepared in 100%. ethanol. After 24 h of incubation, the absorbance at 620 nm was read with a spectrophotometer (Fluostar Omega, Champigny s/Marne, France) to measure the bacterial growth. For each substance, the MIC was defined as the lowest concentration that resulted in null absorbance after 24 h of incubation. Absorbance was represented as % compared to the control strain (set at 100%), and null absorbance was considered when this percentage was below 3%.

For, Prop, But, Hexa, Octa, Cit, Sor and Ben were tested in 6 serial dilutions (2-fold dilutions), from 100 mM to 3.13 mM, while Thy, Car, Van, Eug, Cin, Ger, aPin, Euc, Men, Lin, Limo, Van, Octa, Deca and Dod were tested in 6 serial dilutions (2-fold dilutions), from 7.5 mM to 0.23 mM. The selection of the range of dilution was decided according to previous results in MIC tests run against other terrestrial livestock pathogens in vitro [21,23]. Each test was performed in triplicate.

### 2.4. Minimal Bactericidal Concentration (MBC) Assay

The minimal bactericidal concentration (MBC) test was performed for those compounds that showed an MIC value among the tested concentrations (Hexa, Octa, Sor, Ben, Octa, Deca and Dod among OA; Thy, Car, Van, Eug, Cin, Ger and Van among NIC). The MBC was defined using the same broth microdilution method in 96-well microtiter plates described for MIC assay (10^5^ CFU/mL of bacterial strains incubated in TSB + NaCl with the tested substances); after 24 h of incubation, the 620 absorbance was read at the spectrophotometer in order to confirm MIC values. Samples taken from limpid wells were plated on TSB + NaCl agar plates and the lowest concentration able to avoid the recovery of colonies was defined as the MBC.

### 2.5. Statistical Analysis

The experiments were performed in triplicate for each substance, and the values presented are the means ± standard error of the mean (SEM). MIC data were analyzed with one-way ANOVA followed by the Tukey post hoc test, and differences were considered significant at *p* ≤ 0.05. Each substance tested with its serial dilution was analyzed versus the control strain. The blank of each tested substance was subtracted to the raw optical density (OD) value before analysis.

## 3. Results

### 3.1. Minimal Inhibitory Concentration and Minimal Bactericidal Concentration of OA and NIC against V. anguillarum

**Figure 3:**
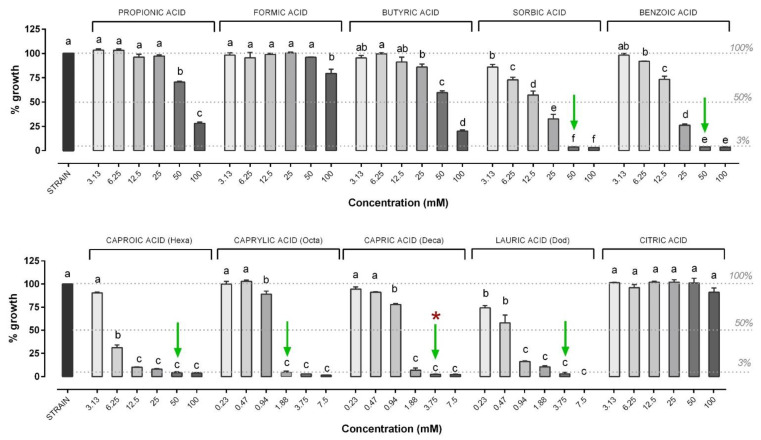
Growth of *V. anguillarum* strain after 24 h of incubation with the different organic acids (OA). *V. anguillarum* growth is expressed as a percentage relative to control (strain), and values are presented as the mean ± standard error of the mean (SEM) (*n* = 3). Data within each OA were analyzed with one-way ANOVA (including the strain value) and columns with different letters were significantly different (*p* ≤ 0.05). Concentrations of each compound are reported in the X axis, while the percentage of growth in the Y axis is reported compared to control. The dashed lines indicate (from top to bottom): control strain level (set as 100% of growth), 50% of growth compared to control (possible OA concentrations able to inhibit 50% bacteria) and the level below which the doses of OA were considered to inhibit bacterial growth (3%). Green arrows indicate minimal inhibitory concentrations (MIC), while red asterisks indicate the minimal bactericidal concentration (MBC) value. When not present, MBC was higher than the highest concentration tested (≥7.5 mM or ≥100 mM). The MBC was defined only for those compounds that showed an MIC value among the tested concentrations.

The results of the in vitro antimicrobial activity of the different OA are shown in Figure 3. In particular, the most effective OA were caprylic (MIC: 1.88 mM), capric (MIC: 3.75 mM) and lauric (MIC: 3.75 mM) acid, followed by sorbic (MIC: 25 mM), benzoic (50 mM) and caproic acid (50 mM). Capric acid was the only OA showing an MBC value against *V. anguillarum* (3.75 mM) within the tested concentrations. SCFA (propionic, formic, butyric acid), as well as citric acid, failed to inhibit the growth of *V. anguillarum* at the tested concentrations.

**Figure 4:**
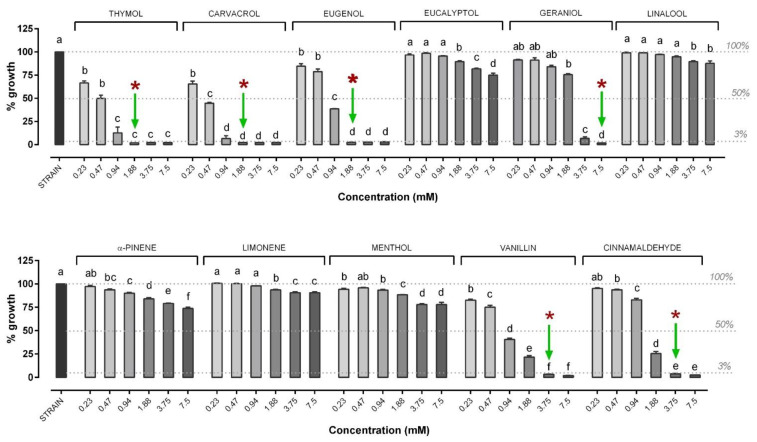
Growth of *V. anguillarum* strain after 24 h of incubation with the different nature-identical compounds (NIC). *V. anguillarum* growth is expressed as a percentage relative to control (strain), and values are presented as the mean ± SEM (*n* = 3). Data within each NIC were analyzed with one-way ANOVA (including the strain value) and columns with different letters were significantly different (*p* ≤ 0.05). Concentrations of each compound are reported in the X axis, while the percentage of growth in the Y axis is reported compared to control. The dashed lines indicate (from top to bottom): control strain level (set as 100% of growth), 50% of growth compared to control (possible NIC concentrations able to inhibit 50% bacteria) and the level below which the doses of NIC were considered to inhibit bacterial growth (3%). Green arrows indicate MIC while red asterisks indicate the MBC value. When not present, MBC was higher than ≥7.5 mM. The MBC was defined only for those compounds that showed an MIC value among the tested concentrations.

Regarding NIC, results are shown in Figure 4 for in vitro antimicrobial activity against *V. anguillarum*. In particular, the most effective NIC were the terpenes thymol (MIC: 1.88 mM), carvacrol (MIC: 1.88 mM), eugenol (MIC: 1.88 mM), geraniol (MIC: 7.5 mM) and the terpenic aldehydes vanillin and cinnamaldehyde (MIC: 3.75 mM). All these MIC values turned out to also be MBC values. Eucalyptol, linalool, menthol, alpha-pinene and limonene failed to inhibit the growth of *V. anguillarum* at the tested concentrations.

### 3.2. Minimal Inhibitory Concentration and Minimal Bactericidal Concentration of OA and NIC against V. harveyi

**Figure 5:**
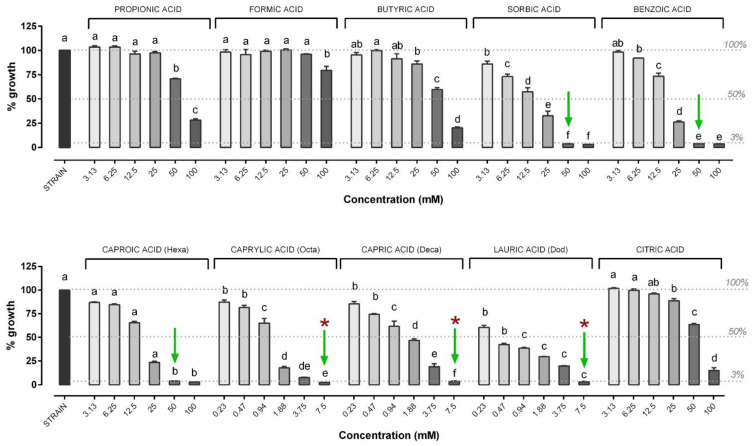
Growth of *V. harveyi* strain after 24 h of incubation with the different OA. *V. harveyi* growth is expressed as a percentage relative to control (strain) and values are presented as the mean ± SEM (*n* = 3). Data within each OA were analyzed with one-way ANOVA (including the strain value) and columns with different letters were significantly different (*p* ≤ 0.05). Concentrations of each compound are reported in the X axis, while the percentage of growth in the Y axis is reported compared to control. The dashed lines indicate (from top to bottom): control strain level (set as 100% of growth), 50% of growth compared to control (possible OA concentrations able to inhibit 50% bacteria) and the level below which the doses of OA were considered to inhibit bacterial growth (3%). Green arrows indicate MIC while red asterisks indicate 5 mM or ≥100 mM). The MBC was defined only for those compounds that showed an MIC value among the tested concentrations.

The results of the in vitro antimicrobial activity of the different OA are shown in Figure 5. In particular, the most effective OA were caprylic (MIC: 7.5 mM), capric (MIC: 7.5 mM) and lauric (MIC: 7.5 mM) acid, for which the MIC values also corresponded to MBC. They were followed by sorbic (MIC: 50 mM), benzoic (MIC: 50 mM) and caproic acid (MIC: 50 mM), which were able to inhibit growth but not have a bactericidal effect. SCFA (propionic, formic, butyric acid), as well as citric acid, failed to inhibit the growth of *V. harveyi* at any tested concentrations.

**Figure 6:**
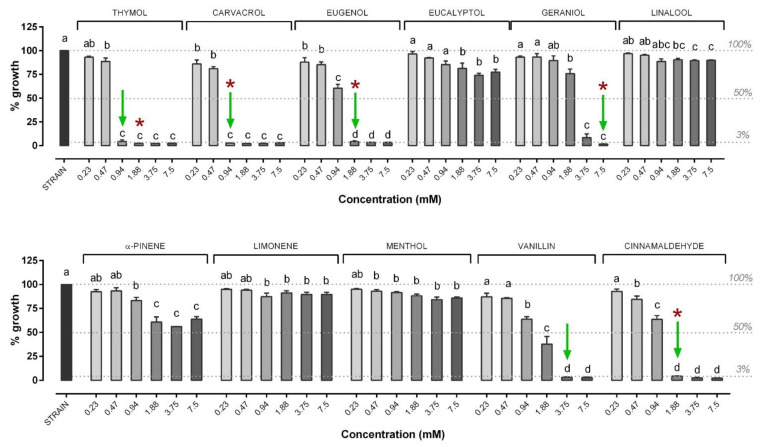
Growth of *V. harveyi* strain after 24 h of incubation with the different NIC. *V. harveyi* growth is expressed as a percentage relative to control (strain), and values are presented as the mean ± SEM (*n* = 3). Data within each NIC were analyzed with one-way ANOVA (including the strain value) and columns with different letters were significantly different (*p* ≤ 0.05). Concentrations of each compound are reported in the X axis, while the percentage of growth in the Y axis is reported compared to control. The dashed lines indicate (from top to bottom): control strain level (set as 100% of growth), 50% of growth compared to control (possible NIC concentrations able to inhibit 50% bacteria) and the level below which the doses of NIC were considered to inhibit bacterial growth (3%). Green arrows indicate MIC, while red asterisks indicate the MBC value. When not present, MBC was higher than ≥7.5 mM. The MBC was defined only for those compounds that showed an MIC value among the tested concentrations.

Regarding NIC antimicrobial activity against *V. harveyi*, results are shown in Figure 6. In particular, the most effective NIC were the terpenes thymol (MIC: 0.94 mM), carvacrol (MIC: 0.94 mM), eugenol (MIC: 1.88 mM), geraniol (MIC: 7.5 mM) and the terpenic aldehydes cinnamaldehyde (MIC: 1.88 mM) and vanillin (MIC: 3.75 mM). Eucalyptol, linalool, menthol, alpha-pinene and limonene failed to inhibit the growth of *V. harveyi* at the tested concentrations.

### 3.3. Inhibition Dose Response Curve of OA and NIC against V. harveyi and V. anguillarum

**Figure 7:**
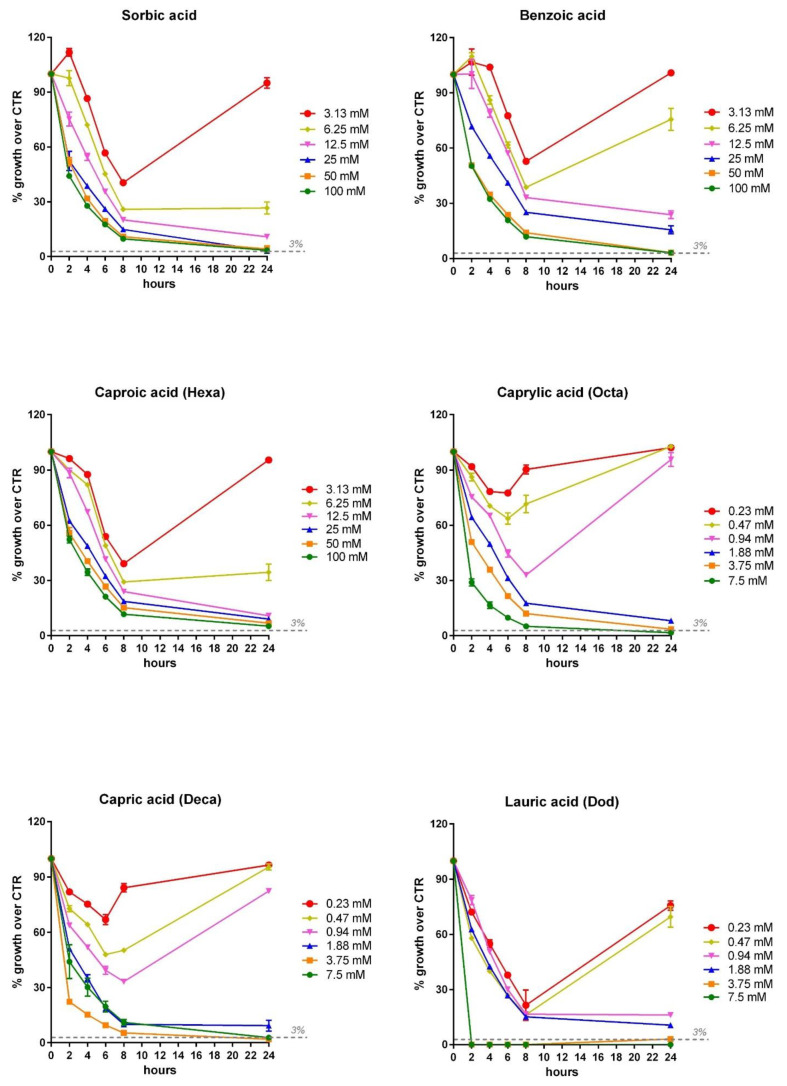

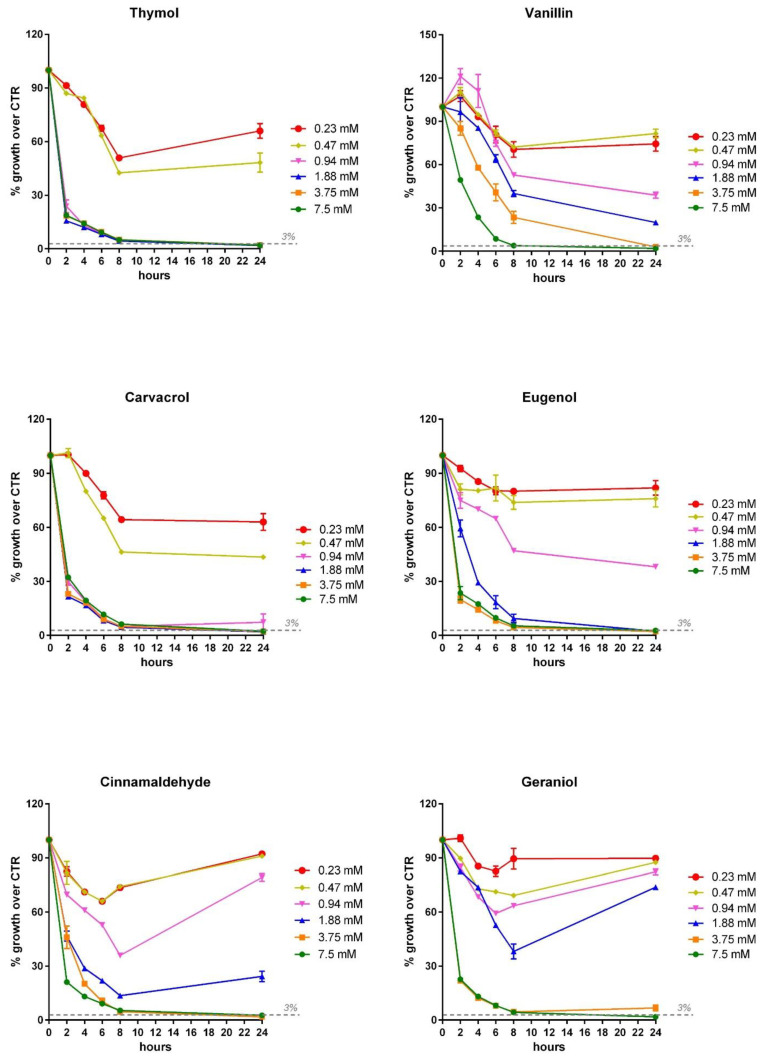
Inhibition dose–response growth of *V. anguillarum* strain following 2, 4, 6, 8 and 24 h of incubation with the different OA and NIC that showed MIC values at 24 h. *V. anguillarum* growth is expressed as a percentage of growth relative to control strain and values are presented as the mean ± SEM (*n* = 3). Each concentration of OA or NIC is represented with a different color, as shown in the legend close to each graph.

**Figure 8:**
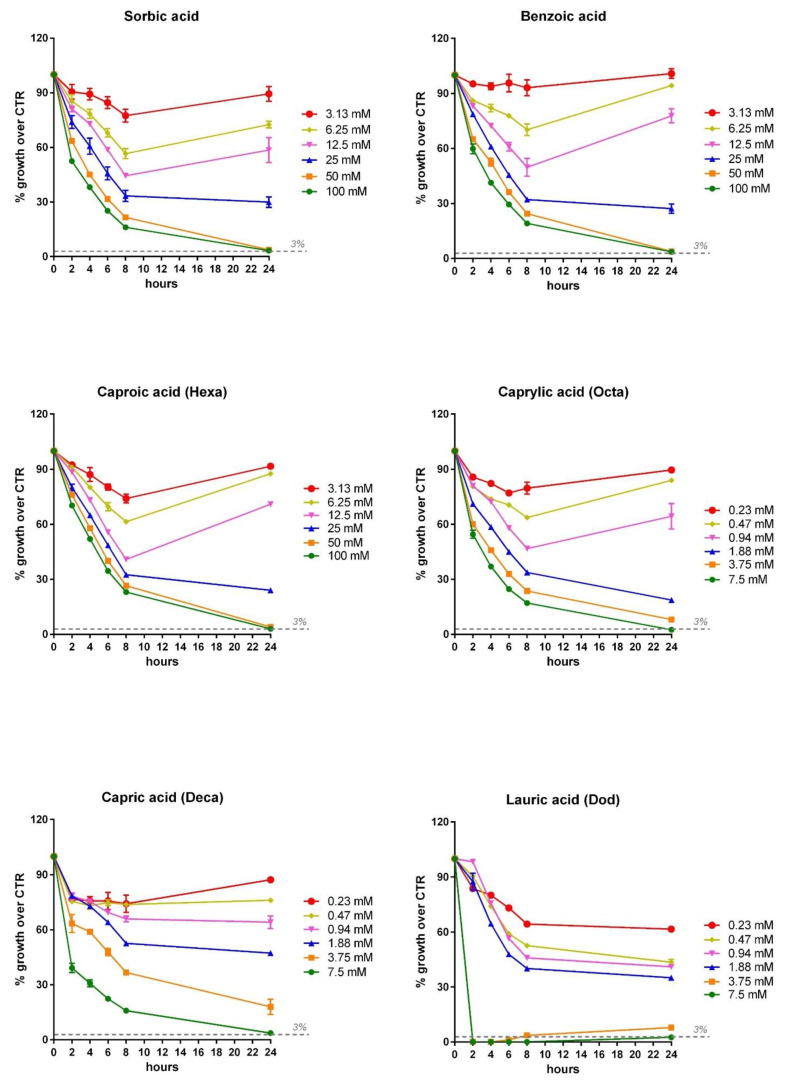

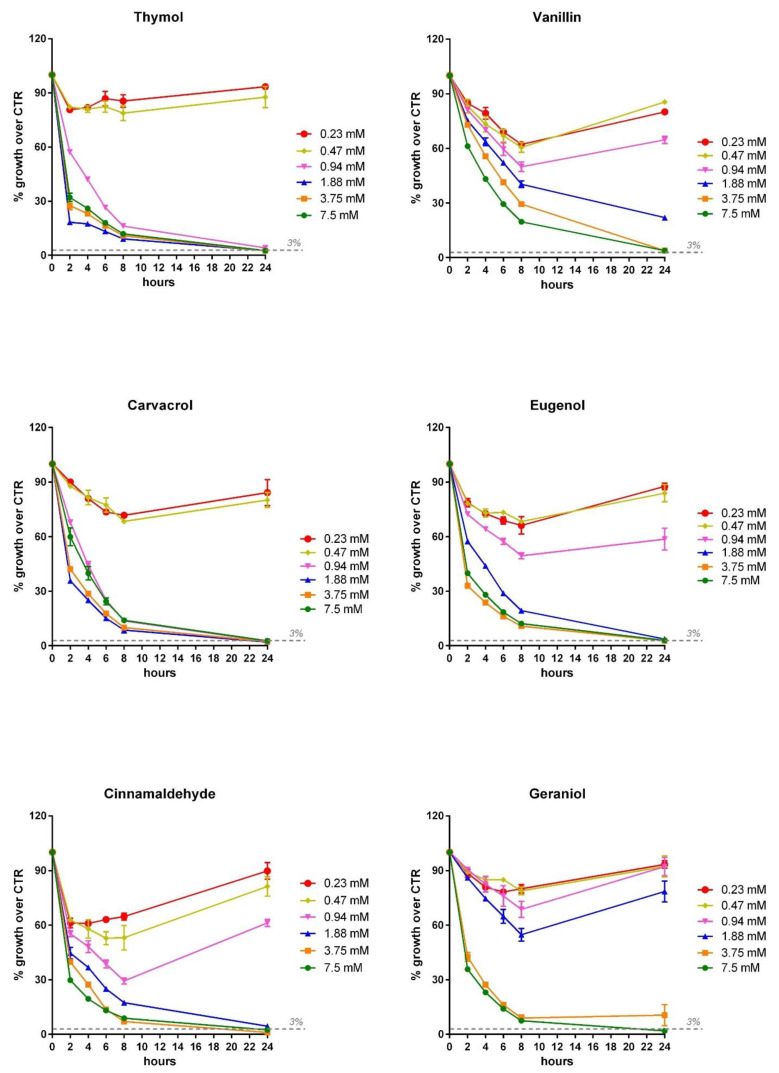
Inhibition dose–response growth of *V. harveyi* strain following 2, 4, 6, 8 and 24 h of incubation with the different OA and NIC that showed MIC values. *V. harveyi* growth is expressed as a percentage of growth relative to control strain and values are presented as the mean ± SEM (*n* = 3). Each concentration of OA or NIC is represented with a different color, as shown in the legend close to each graph.

The two *Vibrio* strains were incubated with all the OA and NIC for 24 h and the OD was read at 2, 4, 6, 8 and 24 h. In Figure 7 (*V. anguillarum*) and Figure 8 (*V. harveyi*), the inhibition dose response curve for all those compounds that showed an MIC value at 24 h (the curves for the other OA and NIC are not shown) is reported. These curves allow for the evaluation of the effect of the different concentrations of OA/NIC on the growth of *V. harveyi* and *anguillarum* at different time points, highlighting how the MIC doses were effective in inhibiting the growth of *Vibrios* over the 24 h of incubation. While the doses lower than MIC’s values were able to reduce the growth in the first hours, they were not able to inhibit the growth of *Vibrios* over 24 h.

## 4. Discussion

*Vibrio* spp. are the causative agents of vibriosis, one of the main diseases in aquaculture farming that is particularly devastating to this business [24]. The indiscriminate usage of a wide spectrum of antibiotics in the past, as well as the metaphylactic and therapeutic use that are still commonplace, have led to an emergence of antibiotic resistance in various bacterial pathogens associated with fish diseases [25], including several *Vibrio* spp. [26]. Among Vibrios, there are many reports about AMR in *V. harveyi* and *V. anguillarum*, two species responsible for a devastating threat to the larviculture and aquaculture industries around the world [27,28]. The aim of this study was to perform a preliminary in vitro screening of a wide panel of OA and NIC (single bioactive compounds of botanicals) in order to detect the molecules with the greatest antimicrobial activity against these 2 *Vibrio* spp. To our knowledge, there are very few studies on the antimicrobial effect of pure single OA or NIC against *Vibrio*, and they do not cover the wide panel of bioactives screened in this study. Although chemotherapy has been widely used to prevent and treat disease outbreaks [29] up until now, the AMR threats, as well as the increasing consumer awareness of the need to reduce antibiotic usage [30], are leading to a worldwide effort to change toward more sustainable aquaculture production methods. In terrestrial livestock, OA and botanicals are two of the most interesting antimicrobial alternatives to antibiotics [18,19]. In the last decade, there has been a growing interest in their usage as feed additives in aquaculture as well. In fact, many in vivo trials were performed to evaluate their effects in terms of growth performance and pathogens load control [25,29,31]. By contrast, there are fewer in vitro studies aimed at broadening the knowledge of the specific antimicrobial activity of these compounds against fish/shrimp pathogens. More precisely, to our knowledge, there are no in vitro studies that have screened the activities of a wide panel of pure OA and NIC against *V. harveyi* or *V. anguillarum*. Not all OA and NIC have the same antimicrobial activity. For this reason, different classes of OA and NIC were analyzed in the present study. The range of concentration tested was decided according to previous results in vitro with other aquatic [32] or terrestrial livestock pathogens [21,23,32], and when it was possible to compare with inclusions tested in vivo [25], we ensured that these doses were included in the range of dilutions tested. Several in vivo studies combine plant extracts or blend of OA/NIC [25,29], so it is often difficult to attribute a specific effect to a specific molecule. For this reason, the purpose of the screening in this study was to determine the specific antimicrobial activity of pure compounds. Our results showed that *V. harveyi* and *anguillarum* were sensitive to the same OA or NIC to the same extent, and this is probably related to a similar pattern of sensitivity of *Vibrio* spp. to the same classes of molecules. Furthermore, we reported sensitivities that are comparable to what was observed in other Gram-negative pathogens, namely *S. typhimurium* and *E. coli* [21,23]. In fact, the most effective molecules in inhibiting *Vibrio* spp. growth were sorbic and benzoic acid among OA and thymol, carvacrol and eugenol among NIC. Even the two aldehydes, vanillin and cinnamaldehydes, which were able to inhibit the growth of *V. harveyi* and *V. anguillarum*, were reported in other studies as capable of exerting antimicrobial action against other Gram-negative pathogens [33]. These molecules have demonstrated the highest antimicrobial activity against several pathogens [18,19], and this might be due to their mechanism of action that is related to the pKa value (anion model) in the case of OA [19], and to the ability, in the case of NIC, to penetrate and disaggregate membrane due to their aromaticity, as well to inhibit quorum sensing and biofilm formation [20,34,35,36]. Nonetheless, in this study, we reported an unexpected susceptibility to MCFA that is generally less or not effective against Gram-negative bacteria [19]. Moreover, we also found how MCFA were the only OA in this study that were not only able to inhibit the growth of *V. harveyi* and *anguillarum*, but also to show a bactericidal effect. To our knowledge, MCFA effects in aquaculture have been poorly explored, but the few studies that are available [37,38] confirmed our results. Nevertheless, some variability across studies is to be expected due to the lack of consistency of the methods used or the comparison between in vivo and in vitro [37,38].

Generally, the most frequently tested OA in vivo are mainly SCFA and citric acid [25]. None of these had strong inhibitory properties against *Vibrio* in our study, unless they were used at the highest concentration (>100 mM), and this is due to the fact these OA need high inclusions to be effective in terms of performance and pathogen load control in aquaculture [25], as well as in terrestrial livestock species [19]. 

Concerning botanicals, studies are mostly performed with plant extracts (powder extracts, essential oils) [29,31] that are not always chemically defined as they are constituted by a variable number of different compounds. This makes it difficult to compare the inclusion and the results obtained; moreover, this aspect highlights the importance of testing the antimicrobial activity of pure single molecules and defining the real players in the antimicrobial mechanism of action.

This study was meant to be a first screening of different OA and NIC, covering a wide range of concentration with the aim to define the antimicrobial activity of pure single compounds that are generally tested in blends or that comprise plant extracts where their concentration is variable or unknown. These results represent the starting point for a further definition of a targeted feed additive aimed at controlling vibriosis in aquaculture, although careful consideration should be afforded to how to transition in vitro results to in vivo. Further analyses will be addressed to broadening the knowledge of the precise mechanism of action behind the antimicrobial effect of those OA/NIC that showed inhibitory and/or bactericidal activity against *V. harveyi* and *anguillarum*.

## Data Availability

Not applicable.
